# Low adherence to Option B^+^ antiretroviral therapy among pregnant women and lactating mothers in eastern Tanzania

**DOI:** 10.1371/journal.pone.0212587

**Published:** 2019-02-22

**Authors:** Kamonga M. Zacharius, Namanya Basinda, Karol Marwa, Emmanuel H. Mtui, Albino Kalolo, Anthony Kapesa

**Affiliations:** 1 Family Health International, Dar es salaam, Tanzania; 2 School of Public Health, Catholic University of Health and Allied Sciences, Mwanza, Tanzania; 3 Department of Pharmacology, Catholic University of Health and Allied Sciences, Mwanza, Tanzania; 4 Department of Epidemiology and Biostatistics, Kilimanjaro Christian Medical University, Moshi, Tanzania; 5 Department of Community Medicine, St. Francis University of Health and Allied Sciences, Ifakara, Tanzania; National Institute of Health, ITALY

## Abstract

**Background:**

Adherence to option B+ antiretroviral treatment (ART) is vital to a successful implementation of Prevention of Mother to Child Transmission (PMTCT) program. Further studies show that optimal viral suppression is also crucial for a successful PMTCT program, however barriers to adherence exist and differ among populations and particularly within few years of its adoption in Tanzania. This study therefore aimed at investigating the level and predictors of adherence to ART option B+ among pregnant and lactating women in rural and urban settings of eastern Tanzania.

**Methodology:**

A cross-sectional study was conducted among 305 pregnant women and lactating mothers on Option B^+^ regime from six health facilities located in rural and urban settings in Morogoro region in eastern Tanzania. Data were collected using a structured questionnaire. Data analysis was performed using descriptive statistics, as well as bivariate and multivariate logistic regression.

**Results:**

Good adherence to option B+ PMTCT drugs was 26.3% and 61.1% among respondents residing in urban and rural areas respectively. The rural residents were 4.86 times more likely to adhere compared to their counterparts in an urban area (aOR = 4.86; 95% CI = 2.91–8.13). Similarly, women with male partners’ support in PMTCT were 3.51 times more likely to have good adherence than those without (aOR = 3.51, 95% CI = 1.21–10.15). Moreover, there was a significantly lower odds of adherence to option B+ among those who had been on treatment between one to two years as compared to those had less than one year of treatment (aOR = 0.45; 95%CI = 0.22–0.93).

**Conclusion:**

Adherence to PMTCT option B+ antiretroviral drugs treatment among pregnant women and breastfeeding mothers was low and much lower among urban residents. Adherence was significantly predicted by rural residence, male partner support and short duration on ART. Efforts to improve adherence should focus on increasing male participation on PMTCT, tailored interventions to urban residents and those who have been on ART for a long duration.

## Introduction

Prevention of mother to child transmission (PMTCT) is a pillar of reduction and elimination of HIV vertical transmission. Considerable efforts have been made with local and international community to design appropriate interventions to eliminate HIV transmission to children but still there are some challenges existing [1]. The use of antiretroviral drugs for PMTCT worldwide has resulted to a marked decrease of Mother to child transmission (MTCT) from over 370000 children in 2009 to as low as 160,000 children in 2014 [2, 3]. Tanzania also responded considerably well through introducing her elimination plan of mother to child transmission aiming at improving health of parents and their children by scaling up comprehensive PMTCT and pediatric HIV care through intensive treatment and supporting services [4].

Since the inception of Implementation of PMTCT services in Tanzania in 2000’s, several milestones have been witnessed ranging from single dose niverapine (sdNVP) during perinatal to the current combination treatment, option B+ [5]. The Option B^+^ PMTCT program in Tanzania was first adopted in September 2013 involving the provision of a lifelong triple antiretroviral drugs to pregnant women as soon as diagnosed [5, 6]. By 2014, more than 90% of all reproductive and child health clinics in Tanzania had started providing PMTCT integrated with reproductive and child health services [5, 7].

Despite of all these underway initiatives, it is well known that a higher level of adherence is desirable for maximum viral load suppression hence prevention of mother-child transmission [8, 9]. Studies have shown that, pregnant women with good adherence to ART have a low risk (less than 5%) of transmitting of HIV infection to their children [9, 10]. Despite of the increasing coverage of elimination of MTCT in Tanzania, there is limited information focusing on the level of option B+ adherence and its covariates in the context rural and urban settings among pregnant women and breastfeeding mothers on treatment [11]. Therefore, this study aims at providing an insight on the current magnitude of adherence to Option B+ antiretroviral drugs intervention and the linked factors in eastern Tanzania.

## Material and methods

### Study design

This was analytical cross-sectional study involving pregnant and lactating mothers on Option B+ attending reproductive and child health clinics in selected health facilities. The study was conducted from October 2017 to December 2017.

### Study area

This study was conducted at six selected health facilities from urban and rural settings of the Morogoro region in eastern Tanzania. The selected health facilities were in Kilosa District (rural) and Morogoro Municipal (urban). The population of the respective districts were 438,175 and 315,866 respectively. Kilosa has a total of 77 health facilities whereas 51 are providing PMTCT services while Morogoro Municipal council has 68 health facilities of whom 48 are providing PMTCT services. Similarly, by 2016 HIV prevalence among the 15–49 age group in the region was 4.2% with an overall viral load suppression of 45.3% among people living with HIV on treatment [12]. Therefore to ensure inclusiveness of the targeted population, the study was conducted in Reproductive and Child Health clinics (RCHC) with highest number of clients and offering the following services: routine antenatal care; child growth monitoring; vaccination, family planning; HIV counselling and testing for pregnant women; PMTCT option B^+^; care for HIV positive women and their infants. These health facilities serve large number of antenatal attendees in RCHC per year ranging in from a minimum of 400 to a maximum of 1000 visits. From urban Morogoro three health facilities were selected; Mazimbu hospital, Sabasaba and Mafiga health centers while in Morogoro Rural, St Kizito Hospital, Kimamba and Kidodi health centres were included in the study.

### Sampling and sample size

Using the adherence of 73.5% to antiretroviral therapy during and after pregnancy as reported by Nachega and his colleagues [13], this study recruited 305 participants as the minimum sample size as calculated from Kish Leslie formula (1965) [14]. Convenient sampling was applied among pregnant mothers and lactating women consulting for PMTCT services at the RCHC. Study participants were serially enrolled until sample size was reached. This was purposely done due to a small number of pregnant and lactating mothers on option B+ treatment. On the day of data collection, mothers were approached as they were arriving and asked to participate freely to the study until the sample size was reached. Patients visiting PMTCT clinic on their scheduled visits were informed about the study. Those patients who fulfilled inclusion criteria and agreed to participate in the study were provided with a consent form for signing.

### Data collection tools and procedures

Data were collected using questionnaire adopted from the tool to measure ART adherence in resource constrained settings developed and validated in South Africa [15]. The questions were translated into Swahili then back translated into English to ensure the consistency and accuracy of the questions. The questionnaire was pretested with fifty HIV positive pregnant women around non-controlled area before the actual data collection in order to ensure the appropriateness of the content regarding the questions, language and organization. Data were collected from pregnant women and lactating mothers who verbally consented and signed the consent form.

### Measurement of variables

Measurement of good or poor adherence were based on pill count method as well as validated set of questions.

Good adherence by pill count: This was considered when the study participant had an adherence of more than 95% of the prescribed pills while poor adherence was when one missed more than 5% of all doses in a month period. Pill count as a measure of adherence was ascertained by counting the number of remaining pills in relation to total number of prescribed doses during last visit.

Adherence by self-reporting method: The level of adherence was also measured using four measurement questions adapted from the experience in South Africa, which were designed to measure adherence in resource constrained settings [15]. A study participant was considered to have good adherence if she responded “no” to all four of the questions. However, if she responded “yes” to at least one question, she was considered to have poor adherence.

Covariates of this study involved social demographics, HIV stigma, adherence counseling, disclosure status, and distance from health facility. Others were level of education, gestation age, and lactation status, and place of residence (rural or urban). Moreover, distance from home to nearby health facility, knowledge on option B+ PMTCT and male partners support were also included.

Knowledge of women on option B^+^ PMTCT was measured from six knowledge assessment questions, with a minimum score of 0 and maximum of 6. The score was categorized into 0–3 and 4–6 for low and high level of knowledge respectively.

Regarding assessment on male partner involvement on adherence to ART Option B^+^, nine questions were used to assess with score of 0 to 9 as minimum and maximum respectively. Male involvement was considered good for those who had a score of 7 and above, moderate for those who had score of 4–6, and low for those who had score of 3 and below. Reasons for poor adherence to PMTCT option B+ were as well explored.

### Data analysis

Data from the questionnaires were entered, cleaned in excel^®^ window 10 and analyzed using STATA^®^ 13 (Stata Corp. LP 2013). Categorical data were summarized using frequencies and their respective percentages. Pearson Chi-square was used to determine the association between the reported pill-count adherence and self-reported adherence. Bivariate binary logic regression was done to determine the crude odds ratio for pill-count adherence for each individual factor. Multivariable binary logistic regression was employed to determine adjusted odds ratio of pill count adherence. Backwards stepwise logistic regression was employed with a cut-off significance level of 10% to obtain a multivariate model. PMTCT knowledge and disclosure of HIV status to male partners were included in the model based on literature evidence [11]. Logistic regression was also used to test for interaction between different covariates. Five (5) independent variables that includes partner education level, place of residence, Time on ART, disclosure to male partner and PMTCT Knowledge were retained in the final logistic model. Variance inflation factor (VIF) was used to determine multicollinearity between exposure variables. Multicollinearity was considered when VIF was more than 10, however there was no multicollinearity between the covariates. Results were considered statistically significant when the p-value was <0.05.

### Ethical considerations

Ethical approval was provided by the Bugando Medical Centre and the Catholic University of Health and Allied Sciences joint ethics and review committee (CREC/246/2017). All respondents of the study signed a consent form before voluntary participation. Permission to carry out the study was as well sought from responsible local authorities.

## Results

### Social demographics and some baseline obstetric characteristics of the study participants

A total of 305 eligible participants were interviewed from all the sampled facilities. About half of them (52.1%) were aged between 26 to 35 years with the other half evenly distributed above and below this age range. Of all study respondents, 61.3% participants were married whereas approximately two thirds (66.2%) had attained primary education. About half of the participants (50.5%) were unemployed (housewives) while almost half of the participants (48.9%) were living in the rural and only 24.3% were pregnant. Of all pregnant women 63.5% were either in 1^st^ or 2^nd^ trimester gestation. Majority of the participants (77.0%) had a parity of less than 4 children. Nearly half of the participants (48.5%) were on option B+ treatment for more than two years (Table 1).

**Table 1:** Social demographics and baseline characteristics of pregnant women and lactating mothers attending option B+ care and treatment clinics in eastern Tanzania.

Variable	Total interviewees (N = 305)	Good Adherence (%)
**Age**		
16–25	64	28 (43.8)
26–35	159	72 (45.3)
36–45	82	32 (39.0)
**Marital status**		
Single	97	34 (35.1)
Married	187	89 (47.6)
Divorced/widowed	21	9 (42.9)
**Education level**		
No education	36	18 (50.0)
Primary education	202	88 (43.6)
Secondary or higher	67	26 (38.8)
**Partner's Education level**		
No education	11	8 (72.7)
Primary education	187	88 (47.1)
Secondary or higher	101	33 (32.7)
**Occupation**		
Unemployed	154	74 (48.1)
Informal sector	122	45 (36.9)
Formal sector	29	13 (44.8)
**Partner's Occupation**		
Unemployed	32	20 (62.5)
Informal sector	217	91 (41.9)
Formal sector	50	18 (36.0)
**Residence**		
Urban	156	41 (26.3)
Rural	149	91 (61.1)
**Pregnancy or Breastfeeding**		
Pregnant	74	35 (47.3)
Breast feeding	231	97 (42.0)
**Gestational age (n = 74)**		
Trimester 1&2	47	20 (42.6)
Trimester 3	27	15 (55.6)
**Parity**		
Less than 4	235	98 (41.7)
4 and above	70	34 (48.6)
**Time on ART**		
<1 year	76	39 (51.3)
1–2 Year	81	29 (35.8)
>2 years	148	64 (43.3)
**Distance from health facility**		
<5 km	178	83 (46.6)
5–10 km	102	37 (36.3)

### Adherence to option B+ antiretroviral treatment

Assessment of pill-count adherence revealed that 43.3% of the participants had a good adherence to option B+ ART. Self-reported adherence based on the 4 questions revealed that 45.9% of respondents had good adherence. Pill count adherence and self-reported adherence were strongly concordant to each other. The overall percentage agreement between pill count and self-reported adherence was 97.4% with the kappa statistics of 0.95 (p<0001).

### Reasons for poor adherence to option B+ antiretroviral treatment

**Fig 1:**
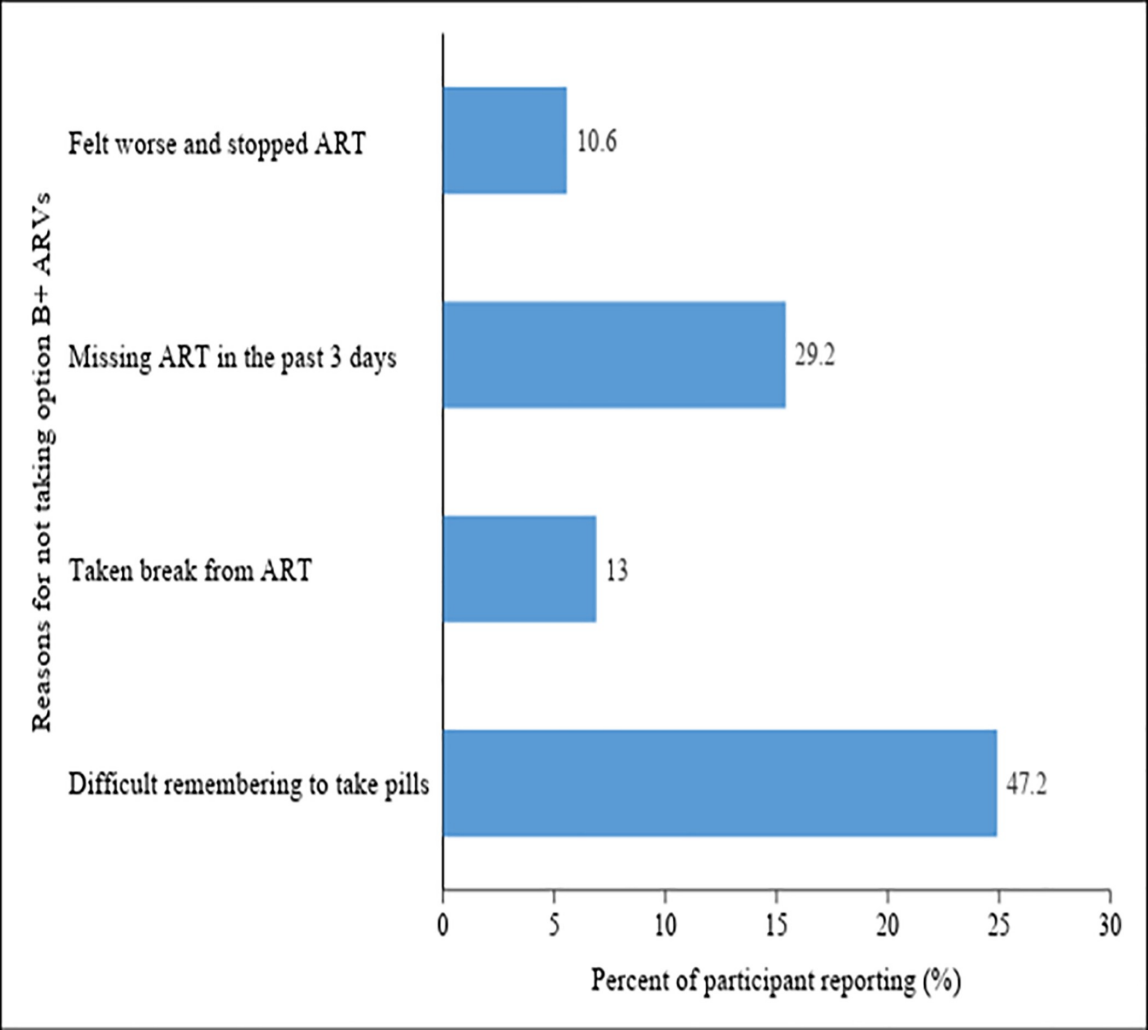
Reasons for not taking the option B+ ARVs for prevention of HIV transmission from mother to child among pregnant women and breast feeding mothers in eastern Tanzania.

Of 161 pregnant women and lactating mothers who had poor adherence, 29.19% of them reported to have missed their ART in 3 days prior to interview as a result of delayed appointment whilst 23.6% took drug holiday due to various underlying reasons. Moreover, approximately half of the participant (47.2%) reported that it was difficult for them to remember take their pills (Fig 1).

### Factors predicting good option B+ ART adherence

During the bivariate logistic regression analysis, the odds of good ART adherence among those whose partners had secondary education or higher were 0.18 times lower compared to those whose partners had no education (OR = 0.18; 95%CI 0.05–0.73; p = 0.02). The married study participants had 1.68 times higher odds of adherence than those who were single (OR = 1.68; 95%CI 1.01–2.79; p = 0.04). Women with partners working in the informal (OR = 0.43; 95%CI 0.20–0.93; p = 0.03) and formal (OR = 0.34; 95%CI 0.13–0.85; p = 0.02) sectors had lower odds of having good ART adherence as compared to women with unemployed partners. Respondents living in rural areas had 4.41 times higher odds of having good ART adherence compared to those living in urban (OR = 4.41; 95%CI 2.71–7.14; P<0.001).

In multivariate logistic regression, place of residence was significantly association with adherence where those who reside in rural areas were 4.86 times more likely to have good adherence as compared to those lived in urban (P<0.001). Those who were moderately supported by their male partners had 3.51 (aOR = 3.15; 95%CI 1.21–10.15; p = 0.02) times higher odds of having good adherence compared to those who had poor male partner support. Moreover, participants who had been on ART for one to two years showed 0.45 times lower odds of good adherence compared to those with less than one year (aOR = 0.45; 95%CI 0.22–0.93; p = 0.03). Table 2 shows the covariates of option B+ adherence

**Table 2:** Tabulation of adjusted and unadjusted factors associated with option B+ ART adherence among pregnant women and lactating mothers attending care and treatment clinics in eastern Tanzania.

Factor	Crude OR	95% CI	P-value	Adjusted OR	95% CI	P-value
**Age**						
16–25	1					
26–35	1.06	0.59–1.91	0.84			
36–45	0.82	0.42–1.60	0.57			
**Marital status**						
Single	1					
Married	1.68	1.01–2.79	0.04			
Divorce/widowed	1.39	0.53–3.63	0.5			
**Education level**						
No education	1					
Primary education	0.77	0.38–1.57	0.48			
Secondary or higher	0.63	0.28–1.44	0.28			
**Partner's Education level**						
No education	1			1		
Primary education	0.33	0.09–1.30	0.11	0.78	0.19–3.18	0.73
Secondary or higher	0.18	0.05–0.73	0.02	0.48	0.11–2.03	0.32
**Occupation**						
Unemployed	1					
Informal sector	0.63	0.39–1.03	0.06			
Formal sector	0.88	0.40–1.95	0.75			
**Partner's Occupation**						
Unemployed	1					
Informal sector	0.43	0.20–0.93	0.03			
Formal sector	0.34	0.13–0.85	0.02			
**Residence**						
Urban	1			1		
Rural	4.41	2.71–7.14	<0.001	4.86	2.91–8.13	<0.001
**Pregnancy/Breastfeeding**						
Pregnant	1					
Lactating	0.81	0.48–1.36	0.42			
**Gestational age (n = 74)**						
Trimester 1&2	1					
Trimester 3	1.69	0.65–4.38	0.65			
**Parity**						
Less than 4	1					
4 and above	1.32	0.77–2.26	0.31			
**Time on ART**						
< 1 year	1			1		
1–2 year	0.53	0.28–1.00	0.05	0.45	0.22–0.93	0.03
>2 years	0.72	0.41–1.26	0.41	0.62	0.33–1.16	0.13
**Distance from health facility**						
Yes	1			1		
No	0.98	0.60–1.60	0.93	1.40	0.61–3.66	0.38
**Partner’s HIV support**						
Poor	1			1		
Moderate	1.60	0.73–3.49	0.24	3.51		0.02
Good	1.34	0.83–2.18	0.23	2.30	0.94–5.60	0.07
**PMTCT Knowledge**						
Poor	1			1		
Moderate	0.87	0.41–1.88	0.74	0.81	0.34–1.95	0.64
Good	0.73	0.35–1.52	0.40	0.67	0.29–1.55	0.35

## Discussion

Antiretroviral therapy adherence level of more than or equal to 95% of all prescribed doses optimizes outcome and minimize occurrence of viral resistance. Overall proportion of ART adherence to option B+ in this study was low, this level is lower than the previously reported by other studies in Sub-Sahara Africa. These studies reported adherence to option B+ among pregnant and lactating ranging from 60% to 87% [16–18]. The current study found more than two times lower odds of good adherence among mother on treatment for 1–2 years as compared to those were on treatment for <1 year. Moreover, mothers/women on treatment for more than two years had a better adherence than those with 1–2 years. And yet, the current study present no significant statistical difference of option B+ adherence between pregnant women and breastfeeding mothers (47% vs 42%). This could be attributed by the fact that some mothers were possibly on option B+ treatment already even before they became pregnant and therefore they were already comfortable with the pills. This implies that mothers who were initiated ART for PMTCT became less motivated to take the pills right after delivery. This finding is lower than that reported by Nachega et al [13] where 73% and 53% well adhered to treatment during antepartum and postpartum periods respectively. However, this review study showed analogously higher adherence during pregnancy as compared to breastfeeding period. Early studies to evaluation option B+ studies in Tanzania reported higher adherence during pregnancy (95%) and lower after two years (during the postpartum (65%) period [11, 19]. Still the current study presents by far lower level of adherence among mothers who were on treatment between 1–2 years (35%) as compared to the previously publish data in Tanzania [19].

Similar low adherence to option B+ in recent studies was observed at tertiary hospital in Uganda among pregnant (52.1%) and lactating women (44.9%) on PMTCT option B+ [20]. Similarly, another published data from the same country found that only 51% of women on option B+ attained good adherence and even lower after delivery [21].

Suboptimal level of adherence found in this study may be contributed by stigma, quality of adherence counselling, male partner support at home, daily life circumstances, and traditional gender norms that might have limited women’s autonomy to access available health resources [22]. Another study conducted in Dar es Salaam, Tanzania, found that lack of motivation after delivery, poverty, and lack of programs that empower women were highly associated to poor adherence to option B+ ART [19]. A qualitative study showed that feeling healthy and adverse effects from ARVs among other reasons explains why women on Option B+ stop taking their medication [23]. The current study however found similar reasons that impended mothers from adhering to treatment.

Area of residence was the most significant factor influencing option B+ adherence followed by male partner support and time on treatment. Other studies found counselling on PMTCT and HIV disclosure as the most significant influencing factors [17, 24]. However, in all these studies male partner support was also found to significantly influence women’s adherence. In Tanzania however, having a treatment supporter in ART services has been also associated with improved clinical outcome [25].

This study found that women residing in rural areas were more likely to have good adherence than those living in urban. On contrarily, other studies found that women living in urban areas adhered more to ART than their counterparts in rural areas [16]. The current finding may be supported by a study done in a neighboring country (Kenya) which found that women living in rural areas describe their lives as being secured and family controlled so becomes easy for them to steal partners support towards PMTCT services [26]. Furthermore, higher attendance to PMTCT services among male partners residing in rural areas than their counterparts in urban areas has been described in Tanzania [27]. These factors may possibly explain why the rural residents as compared to urban had a better uptake of HIV prevention from mother to child, adherence to option B+ ART.

Study strength and limitations: This study has some several strength including presenting option B+ ART adherence in the context of rural and urban viewpoint. Moreover, the use of two methods measuring adherence increased trustworthiness of the findings. On the other hand, this study may have been affected by social desirability and recall bias. Measurement of adherence lacked the plasma concentration of ARVs and therefore ascertaining the drug plasma concentration was not done. Moreover, other factors that might affect the outcome of interest like psychological distress and social capital were as well not explored in this study. Therefore, generalization of the results may be limited to the selected health facilities of eastern Tanzania.

## Conclusion and recommendations

The option B+ adherence level was low and much lower among urban residents as compared to their counterparts in rural areas. The low proportion of good adherence of option B+ for PMTCT in eastern Tanzania might be signalizing a regression of the PMTCT program in eastern Tanzania. Male partner support, time on ART and area of residence were significant predictors of adherence to option B+ treatment. Arraying more efforts to enhance male partner support and involvement and focusing on those on treatment for a longer duration in the PMTCT program may yield more significant outcome. Moreover more efforts to monitor option B+ adherence should be made in urban settings while implementing cohort monitoring and evaluation of the barriers as well as regular viral load measurements.

## Supporting information

S1 File This is data file of 305 pregnant women and breastfeeding mother on option B+ antiretroviral treatment in eastern Tanzania. (DTA)
